# Clinical application of digital technology in bone augmentation: a narrative review

**DOI:** 10.3389/fmed.2026.1852710

**Published:** 2026-06-03

**Authors:** Meng Sicheng, Wang Maoxia

**Affiliations:** 1 West China School of Public Health and West China Fourth Hospital, Sichuan University, Chengdu, China; 2 School of Stomatology, Xinjiang Medical University, Urumqi, China

**Keywords:** bone augmentation, customized bone block, customized titanium mesh, digital workflow, surgical guides

## Abstract

Sufficient bone volume surrounding dental implants is essential for successful implant treatment outcomes. Clinically, a variety of bone augmentation techniques have been utilized to address bone deficiencies. Nonetheless, existing bone augmentation methods remain highly technically sensitive, extended surgical durations, and entail certain risks of complications. The integration of digital technologies with bone augmentation procedures has the potential to streamline and enhance the efficiency of these surgeries. This article provides a comprehensive review of the current applications and research advancements concerning the use of digital technologies in bone augmentation.

## 1 Introduction

Over the years, dental implant therapy has accumulated substantial preclinical and clinical evidence supporting its efficacy. Numerous longitudinal clinical studies have demonstrated that dental implant therapy achieves high success rates, establishing it as a crucial modality for addressing tooth loss (1–4). Adequate alveolar bone volume surrounding an implant is essential for optimal treatment outcomes; insufficient bone volume can adversely affect the three-dimensional positioning of the implant, leading to inadequate initial stability and potentially compromising the aesthetic and functional results of the implant restoration (5–7). However, bone defect is relatively common among patients undergoing implant procedures, with research indicating that approximately 50% of implant sites exhibit such deficiencies (8). In such contexts, surgical intervention aimed at augmenting bone volume is essential to ensure the long-term success of implant therapy. Consequently, a range of bone augmentation techniques, including autogenous bone blocks, guided bone regeneration (GBR), and maxillary sinus elevation, have been extensively utilized in clinical practice for the management of bone defects (5, 9–11). Despite these advancements, current bone augmentation methodologies remain highly technique-sensitive, requiring significant expertise and clinical proficiency from practitioners, as well as extended surgical durations and the potential for complications. The pursuit of more straightforward and effective approaches to bone augmentation surgery represents a critical challenge that demands urgent attention.

In recent years, the integration of digital technology within the field of dentistry has significantly revolutionized traditional treatment methodologies. Specifically, in the domain of dental implantology, digital technologies have been extensively employed in various aspects such as the design and fabrication of prostheses, the creation of digital implant surgical guides, implant navigation, and preoperative planning (12–14). These technologies enable clinicians to accurately simulate postoperative outcomes prior to surgery and facilitate the precise execution of implant procedures, thereby enhancing the predictability of treatment results. Recognizing the distinct advantages conferred by digital technology, researchers have also explored its application in the bone augmentation. This involves utilizing digital tools for the three-dimensional reconstruction of the jawbone, simulating the expected morphology of bone grafts, and subsequently employing 3D printing technology or computer-aided design/computer-aided manufacturing (CAD/CAM) to support the precise execution of bone augmentation procedures (15, 16). Currently, various digital bone augmentation techniques have been applied in clinical settings. This article reviews the current applications and research advancements of digital technologies in bone augmentation.

## 2 Clinical application of digital technologies in bone block grafting

Autogenous bone block grafts have traditionally been considered the “gold standard” for bone augmentation (5, 17). Clinical research has demonstrated that these grafts can effectively repair large-scale bone defects and significantly contribute to the long-term success of implant procedures (10, 18–22). Nonetheless, the utilization of autogenous bone block grafts necessitates the creation of an additional surgical site for bone harvesting, followed by the manual shaping and fixation of the bone block by the surgeon. This approach is highly operator experience sensitive, requires significant time investment, and is associated with potential complications at the donor site, including altered nerve sensation and hematoma formation (23–25). These limitations restrict the widespread clinical application of autogenous bone block grafts.

In order to mitigate the high technical sensitivity and risk of damaging adjacent critical anatomical structures during manual autogenous bone block grafting, De Stavola et al. employed three-dimensional design software to reconstruct a model of the patient’s jawbone. This approach facilitated the calculation of the necessary size of the autogenous bone block based on the extent of the bone defect. The software was used to design osteotomy lines at the donor site while strategically avoiding vital surrounding anatomical structures, and a bone-supported osteotomy guide was generated in accordance with the position of these lines. This methodology enabled precise preparation of the osteotomy lines during surgery, guided by the osteotomy guide (26). In the case report by De Stavola et al. the osteotomy guide demonstrated a precision of 0.25 mm. Additionally, a case series involving 13 patients assessed the safety of employing the osteotomy guide, reporting no occurrences of nerve sensory abnormalities or damage to adjacent teeth following the acquisition of autogenous bone blocks with the guide. This suggests that the osteotomy guide effectively aids in preventing damage to surrounding critical anatomical structures (27). Furthermore, Cristoforetti et al. evaluated the accuracy of the osteotomy guide and found that the deviation in obtaining autogenous bone blocks using this guide was within 0.4 mm (28).

While the osteotomy guide aids surgeons in obtaining autogenous bone blocks with high accuracy and significantly mitigates the risk of injuring adjacent critical anatomical structures, it still necessitates a substantial amount of surgical time for the harvesting and refinement of bone blocks. Consequently, the utilization of allograft, xenograft, and synthetic bone blocks for bone augmentation has increasingly become a focal point of research (29–31). The ease with which these bone substitutes can be shaped renders them highly suitable for applications involving digital technology. In the early stages, Jacotti et al. employed 3D printing technology to fabricate patient-specific jawbone models, allowing for the pre-surgical shaping of bone blocks on these models to produce customized allogeneic bone blocks for bone augmentation (32). Amorfini et al. conducted a randomized controlled trial comparing this bone augmentation technique with the use of particulate bone substitute materials for guided bone regeneration. Their findings indicated comparable volumes of newly formed bone between the two methods after 1 year of functional loading (33). The advent of CAD/CAM technology has significantly streamlined and enhanced the efficiency of processing customized bone blocks. In a concept validation study by Schlee et al. in 2013, involving two patients, the researchers digitally simulated the morphology of bone grafts in the defect area, designed customized bone block shapes accordingly, and utilized CAD/CAM technology to precisely mill allograft bone blocks into these customized configurations. This study documented a horizontal bone gain of 7.18 mm at 7 months postoperatively (34). Subsequently, numerous case reports have corroborated the successful application of customized allogeneic bone blocks in bone augmentation (35–37). A 5-year follow-up case report examined a biopsy from the augmented area 5 years post-operation, demonstrating satisfactory vascularization and the absence of residual allograft material in the bone augmentation region (16). Wang et al. conducted a randomized controlled trial to compare the clinical outcomes of customized allogeneic bone blocks with those of autogenous bone blocks. The findings indicated that customized allogeneic bone blocks resulted in superior bone augmentation outcomes and decreased surgical duration (38).

Due to the limited availability of allograft bone donors, there has been growing interest among researchers in customized xenograft bone blocks and customized synthetic bone blocks. Mirković et al. documented the clinical application of customized xenograft bone blocks through case studies (39). In a separate case report, Figliuzzi et al. discussed the short-term clinical outcomes of CAD/CAM-fabricated hydroxyapatite bone blocks, noting significant new bone formation 6 months following bone augmentation surgery, along with satisfactory functional performance of implants in the augmentation area over a 1-year follow-up period (40). A prospective clinical case series involving 10 patients demonstrated that CAD/CAM-fabricated porous hydroxyapatite bone blocks can effectively reduce surgical time while achieving favorable osteogenic outcomes (41). Similarly, Mangano et al. employed CAD/CAM customized biphasic calcium phosphate blocks for bone augmentation, with histological analysis one year post-operation revealing significant new bone formation in the augmented area (42). It is important to note, however, that current research on customized bone blocks primarily consists of case reports, and more extensive, high-quality clinical evidence is required to substantiate the efficacy of customized bone blocks in bone augmentation.

## 3 Clinical application of digital technologies in guided bone regeneration

Guided Bone Regeneration employs barrier membranes to inhibit the infiltration of soft tissue into bone defect areas. This approach facilitates the preferential entry of osteogenesis-related cells, which migrate more slowly, into the defect, thereby providing a stable environment conducive to new bone formation (43–45). Among the various GBR techniques, the use of absorbable collagen membranes in conjunction with particulate bone substitute materials is prevalent due to their superior biocompatibility and reduced risk of complications, leading to sustained clinical success (5). Nonetheless, the suboptimal mechanical properties of absorbable collagen membranes and particulate bone substitutes pose challenges for practitioners in achieving and maintaining the desired graft shape, ultimately impacting the effectiveness of bone augmentation (46).

In a retrospective cohort study, Wang et al. employed digital technology to simulate the morphology of bone grafts to achieve and maintain optimal bone augmentation morphology in guided bone regeneration. They subsequently designed and fabricated surgical guides for bone augmentation. During surgical procedures, these guides facilitated the shaping of a composite of injectable platelet-rich fibrin (i-PRF) and particulate bone substitute materials, termed i-PRF bone blocks, thus enabling precise control over the bone augmentation form. The study concluded that the application of digital technology in shaping i-PRF bone blocks led to increased bone graft thickness in the coronal portion of the implant upon wound closure (47). Furthermore, in a prospective clinical study, Wang et al. demonstrated that this digital workflow significantly enhanced bone thickness 6 months postoperatively (48). Trace et al. also demonstrated in an *in vitro* study that the application of surgical guides in GBR improves the accuracy of the resulting graft shape (49).

Additionally, the use of titanium mesh in GBR is another strategy to enhance the mechanical properties of bone grafts (50). However, in traditional bone augmentation procedures, surgeons need to trim and shape the titanium mesh intraoperatively, which is technically sensitive and increases surgical time (51, 52). Moreover, the sharp edges of titanium mesh produced during manual trimming and shaping may increase the risk of postoperative wound dehiscence (50, 51, 53). To address these shortcomings of traditional workflows, Al-Ardah et al. performed three-dimensional reconstruction of the jawbone based on preoperative cone-beam computed tomography (CBCT) and conducted virtual tooth arrangement in the bone defect area, thereby simulating the morphology of bone grafts under the guidance of prostheses. Subsequently, they utilized 3D printing technology to print a jaw model containing the simulated bone grafts. Pre-trimming and pre-bending of prefabricated titanium mesh were then performed on this jaw model before surgery to obtain pre-bent customized titanium mesh for GBR (54). Notably, Al-Ardah et al. also achieved precise placement of the pre-bent customized titanium mesh using a retention device similar to a orthodontic clear retainer (54). Zhang et al. indicates that the mean error of this retention device is approximately 0.5 mm (55).

Li et al. investigated the clinical outcomes of pre-bent customized titanium mesh through a retrospective case series study. Their findings revealed that using pre-bent customized titanium mesh resulted in an average vertical bone augmentation of 2.48 mm and horizontal bone augmentation of 4.11 mm. Additionally, pre-bent customized titanium mesh effectively achieved the preoperatively simulated bone augmentation outcomes (56).

Pre-bent customized titanium mesh, as an advanced digital bone augmentation technique, necessitates relatively minimal equipment requirements and is readily applicable in clinical settings. The integration of CAD/CAM technology and 3D printing in the fabrication of these customized titanium meshes has streamlined and enhanced the efficiency of the production process. In 2011, Ciocca et al. conducted simulations of bone graft morphology guided by prostheses and implants, subsequently employing CAD/CAM technology to produce customized titanium meshes for bone augmentation in atrophic maxillae (57). A prospective clinical study involving nine patients demonstrated that the application of CAD/CAM customized titanium mesh resulted in horizontal bone augmentation of 3–4 mm (58). However, it is important to note that six of the nine patients experienced postoperative exposure of the titanium mesh. Additionally, Ciocca et al. reported in a case series that 3D-printed customized titanium mesh achieved vertical bone augmentation of 2.5 mm and horizontal bone augmentation of 3.4 mm in the treatment of maxillary defects (59). In a retrospective case series study, Li et al. examined the outcomes of bone augmentation using 3D-printed customized titanium mesh. Their results indicated that the bone augmentation observed 6–9 months postoperatively closely matched the preoperative simulations of bone graft morphology (15). Furthermore, a separate clinical controlled trial compared the clinical outcomes of 3D-printed customized titanium mesh with those of traditional titanium mesh, demonstrating that the customized approach not only reduced surgical time but also minimized the number of bone screws needed for securing the titanium mesh (60). A meta-analysis has demonstrated that customized titanium mesh is associated with greater horizontal bone gain and a tendency for reduced complications in comparison to conventional titanium mesh in the context of bone augmentation procedures (61).

## 4 Clinical application of digital technologies in sinus floor elevation

Sinus floor elevation is widely utilized aimed at augmenting the available bone height in the posterior maxilla, and it has emerged as one of the most prevalent bone augmentation methods in clinical practice due to its high predictability (9). Nevertheless, anatomical variations associated with the maxillary sinus, such as sinus septa, atypical sinus floor morphology, and intraosseous vessels, can complicate the surgical procedure and elevate the risk of complications (62). To enhance the precision of window positioning during lateral window sinus floor elevation, Mandelaris et al. pioneered the application of digital technology for window position planning in 2008, leading to the design and fabrication of CAD/CAM-milled maxillary sinus window guides for sinus floor elevation (63). In scenarios involving the presence of maxillary sinus septa, Wang et al. employed a dual-window maxillary sinus guide in a case report to circumvent the septa, thereby facilitating safe and precise lateral window sinus floor elevation (64). Kocyiğit et al. investigated the clinical outcomes associated with guided sinus floor elevation, concluding that the utilization of guides facilitated the preparation of the surgical window and mitigated the risk of maxillary sinus membrane perforation. However, this approach was also associated with extended surgical durations and limited visibility of the surgical field (65). A clinical controlled trial further compared the outcomes of guided versus conventional sinus floor elevation, revealing that guided techniques enabled precise window preparation and decreased the risk of membrane perforation, thus presenting a safe alternative to traditional methods (66). Additionally, digital technologies have been shown to enhance the precision of crestal approach sinus floor elevation. Pozzi et al. employed digital implant guides to regulate the depth of implant site preparation and subsequently directed osteotomes for crestal sinus floor elevation, thereby improving the predictability of the surgical procedure (67). Subsequently, Pozzi et al. conducted a prospective cohort study with a follow-up period of 52 months to evaluate the clinical outcomes of guided crestal sinus floor elevation. The study demonstrated that this technique, guided by digital templates, could reduce the incidence of complications, shorten treatment times, and serve as an effective minimally invasive method for sinus floor elevation (68).

## 5 Discussion

Digital techniques in bone augmentation constitute an advanced and innovative area of study within the field of oral implantology, which allow clinicians to simulate postoperative outcomes preoperatively and assist in the precise execution of bone augmentation during treatment. Table 1 provides an overview of digital techniques currently employed in clinical settings for bone augmentation. Noteworthily, the extensive adoption of a novel clinical technique is contingent not solely upon their innovativeness, but also on the interplay of various multidimensional factors, such as the relative advantages, scientific evidence, cost-effectiveness, acceptance by healthcare providers and patients, organizational support, and policy environments (69–73).

**TABLE 1:** Summary of digital techniques in bone augmentation.

Digital technique	References
Bone block grafting	
Osteotomy guide	(26–28)
Customized allogeneic bone block	(32–38)
Customized xenograft bone block	(39)
Customized synthetic bone block	(40–42)
Guided bone regeneration	
Surgical guide to shape the bone grafts	(47–49)
Pre-bent customized titanium mesh	(54–56)
CAD/CAM customized titanium mesh	(57, 58)
3D-printed customized titanium mesh	(15, 59, 60)
Sinus floor elevation	
Surgical guide for positioning maxillary sinus window	(63–66)
Surgical guide for crestal sinus floor elevation	(67, 68)

The relative advantages, including enhancements in efficacy and efficiency, serve as the principal motivators for the adoption of new technologies. In the context of bone augmentation procedures, digital technologies are predominantly employed to achieve precise control over the morphology of bone grafts, thereby reducing technical sensitivity and shortening surgical durations. However, the integration of digital technologies does not necessarily result in a substantial decrease in the technical threshold required for bone augmentation. Critical factors such as flap design, soft tissue management, and others remain essential for the successful execution of bone augmentation procedures. At present, these aspects cannot be entirely supplanted by digital technologies. The success of bone augmentation continues to depend significantly on the surgeon’s expertise and experience. For example, a high rate of titanium mesh exposure has been observed in some clinical studies utilizing customized titanium mesh (58). Furthermore, the majority of contemporary research primarily focuses on the duration of the surgical procedure (38). Conversely, the digital workflow necessitates additional preoperative time for digital simulation and the fabrication of digital products, such as customized titanium meshes, customized bone blocks, and surgical guides, when compared to the traditional workflow. These digital techniques also incur higher costs relative to traditional methods. Further research is necessary to assess the cost-effectiveness of digital techniques. Additionally, existing research on digital bone augmentation techniques is primarily derived from clinical case reports or pilot studies, resulting in insufficient quality of evidence and inadequate long-term outcome data. To support and confirm the findings of existing studies, future research should prioritize the implementation of long-term, large-sample randomized controlled trials or real-world studies.

In summary, digital techniques in bone augmentation demonstrate significant potential that justifies further investigation; however, additional clinical evidence is necessary to substantiate their widespread adoption as a highly reliable approach.
